# Long term water trapping in Pickering emulsions undergoing compositional ripening†

**DOI:** 10.1039/d3sm00856h

**Published:** 2023-11-09

**Authors:** Raj Tadi, Beth Green, Thomas Curwen, Paul S. Clegg

**Affiliations:** a School of Physics and Astronomy, University of Edinburgh Peter Guthrie Tait Road Edinburgh EH9 3FD UK rajtadi95@gmail.com; b Mondelēz International, Reading Science Centre Whiteknights Campus, Pepper Lane Reading RG6 6LA UK

## Abstract

One approach to achieving low-calorie foods is to substitute regions of high-calorie content with water droplets, forming water-in-oil emulsions. However, in complex food systems consisting of multiple species of dispersed phases, compositional ripening may occur in which the emulsified water undergoes mass transfer to droplets filled with a species that is less soluble in the continuous phase, for example sugar. Here we present two model systems and use them to study compositional ripening for water-in-oil Pickering emulsions. Water-in-dodecane and water-in-tributyrin emulsions stabilised by PMMA particles were prepared and combined with similar emulsions that included sugar in the water. We use confocal microscopy as a function of time combined with particle tracking to explore how these systems evolve in time. For dodecane, as the system evolves, the pure water droplets appear to crumple due to the loss of water; in extreme cases, they eventually ‘explode’. Simultaneously, the sugar-filled droplets expand and slowly coalesce. Evidently, our interfacial coating of particles is unable to suppress compositional ripening. In contrast, pure water droplets in tributyrin crumple into small stable structures, potentially retaining water. We show that decreasing the concentration of the sugar solution also decreases the rate of change of water droplet size for both oils. Observations of droplet ‘explosions’ confirm that the driving force can overcome the trapping of the particles at the interface, in contrast to the case of Ostwald ripening. However the crumpled states in the tributyrin system provide some indication that this effect can be overcome.

## Abstract 2

Water-in-oil emulsions are a potential way to achieve low-calorie foods. We investigate compositional ripening in two water-in-oil Pickering systems using confocal microscopy and particle tracking, revealing two contrasting end fates.

## Graphical abstract

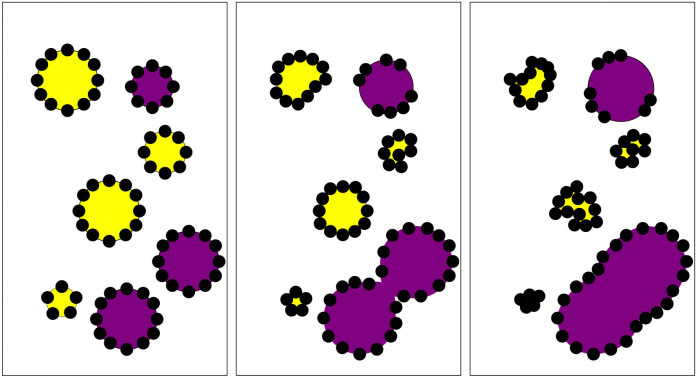

## 1 Introduction

There is a strong desire to make healthier foods by replacing high calorie oil components with water in the form of emulsions.^1^ Food systems are however very complex and heterogeneous causing such emulsions to degrade *via* coalescence, Ostwald ripening, and compositional ripening. In coalescence droplets in contact reduce their total interfacial cost by merging. Ostwald ripening instead results in the growth of large droplets at the expense of small ones, driven by the differences in Laplace pressure, and is a diffusion based mechanism.

To prevent the destabilisation of emulsions *via* coalescence and Ostwald ripening, various surface active species such as surfactants, proteins *etc.* can be used. Specifically, in a Pickering emulsion the droplet surfaces are covered with colloidal particles. The stabilising behaviour of Pickering emulsions is indicated by eqn (1), the energy required to remove an isolated colloidal particle from a flat water–oil-interface.

1 *E*_ads_ = π*r*^2^*γ*{1 ± cos *θ*}^2^

where *r* is the radius of the colloid, *γ* is the interfacial energy of oil/water interface, and *θ* is the contact angle. For typical colloids at the interface these energies can be many orders of magnitude higher than thermal energy, *kT*. As a result they are considered irreversibly adsorbed at the interface. More recently, various food-grade biocompatible colloids have been explored for stabilising emulsions,^2^ such as those derived from polysaccharides and proteins.

Such emulsions have been shown to be stable against coalescence *via* the formation of a dense monolayer forming a physical barrier to overcome. However, it has also been shown that droplets can remain stable without a full monolayer.^3^ Surprisingly, Ostwald ripening can also be suppressed *via* the use of colloidal particles.^4,5^

Emulsion droplets within a food system will often be surrounded by many varied components. If this includes a second dispersed component that is miscible with the contents of the droplets, compositional ripening can occur. This is driven by the tendency for the two species to mix. Compositional ripening is also a diffusion mechanism similar to that of Ostwald ripening, though in the presence of surfactants it can proceed *via* a ‘micelle carrier’ mechanism.^6^

**Fig. 1:**
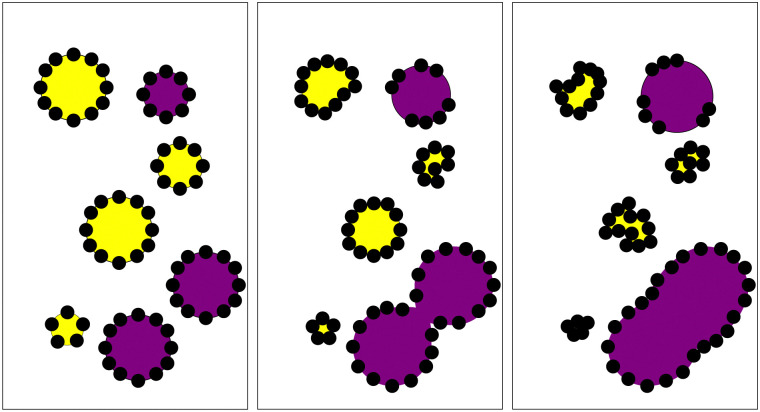
Cartoon of compositional ripening in Pickering emulsion mixtures, with the water droplets (yellow) shrinking while the sugar solution droplets (purple) grow and coalesce. Without the presence of additional colloids, gaps form on the growing droplets' particle layer, potentially leading to their partial coalescence.

Considering two populations of droplets (as shown in Fig. 1) the more soluble species is considered ‘mobile’ while the less soluble is considered ‘immobile’^6^ with the transfer taking place from ‘mobile’ to ‘immobile' droplets. The chemical potential *μ* of the mobile phase is described by:2
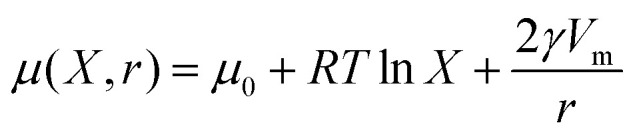
where *X* is the mole fraction, *r* is the droplet radius, *γ* is the oil–water tension, *V*_*m*_ is the molar volume of the mobile phase, and *μ*_0_ is the standard chemical potential corresponding to the pure mobile phase in bulk.

The driving force for mass transfer between droplets is the difference in chemical potential, *μ*, between the two populations of droplets. From eqn (2), we can see that to overcome compositional ripening, one can modify the droplets such that the composition contribution is equal to the Laplace pressure contribution. For example, by changing the droplet sizes such that it matches the chemical potential gradient or *vice versa*. Additionally, adding another solute to the ‘mobile’ droplets could balance the mass transfer. Without modifying these aspects however, it has not been determined whether other aspects of the emulsion can overcome the process. Researchers have overcome compositional ripening by creating a continuous shell which isolates droplets from their environment.^7^

The derivation in Binks *et al.*^6^ following the work by Ugelstad *et al.*^8^ suggests that in addition to the factors in eqn (2), the rate of ripening is affected by the diffusion coefficients and solubilities of the mobile phase in the continuous phase, the number and size of ‘mobile’ and ‘immobile’ droplets, and the difference in chemical potential between the two. In addition, the ripening rate is affected by any possible energy barriers for transport across the oil/water interface.^9^ As shown in Kabalnov *et al.*^10^ the use of soluble and insoluble surfactants modifies the surface tension of the droplet which in turn modifies the chemical potential difference. This may provide a potential route to overcome or suppress compositional ripening. However, it is heavily dependent on the properties of the interfacial layer of colloids.^5^

Here we compare compositional ripening in two mixed Pickering water-in-oil systems (Fig. 1), where the driving force for compositional ripening is provided by the addition of sugar solution droplets. In this scenario we consider the pure water as the ‘mobile’ phase and the sucrose as the ‘immobile’ phase. In the first model system, dodecane is used as the continuous phase, which allows the formation of a dense PMMA monolayer on the oil–water interface. For the second system, tributyrin, a short chain triglyceride found in many foods, was used to replace dodecane as the oil phase as a means of exploring changes in solubility, diffusion, and density as well as the colloid-colloid interactions. This results in two contrasting emulsions with different ripening behaviours. Droplets were observed with confocal microscopy and tracked to determine their changes in size.

## 2 Methods

### 2.1 Materials

The aqueous phases used were water (deionised using a Millipore Milli-Q reagent system with resistivity of 18 MΩ cm) and sugar solutions made from dissolving table sugar (Sainsburys) with deionised water at weight fractions of 10%, 20%, 30%, 40%, 50%, and 64%. The highest concentration of 64% was chosen as this is close to the maximum solubility of sugar in water. Different phases were dyed with sodium fluorescein, rhodamine B and pyranine with weight concentrations around 0.01%. The oils used were *n*-dodecane (Sigma Aldrich 99%) and tributyrin (Sigma Aldrich 99%, melting temperature −75 °C); these were dyed with Nile-Red when required.

Poly(methyl methacrylate) (PMMA) colloids sterically stabilised with poly(12-hydroxystearic acid) (PHSA) hairs were used, dyed with NBD (*r* ≈ 362 nm) and undyed (*r* ≈ 355 nm).

### 2.2 Dispersion preparation

To prepare the continuous phases, dry PMMA particles were dispersed in the dodecane and tributyrin at volume fractions of 1% using a pulsed sonicator probe (Sonics Vibracell). The protocol consisted of a 5 second sonication followed by a 5 second rest, repeated for a total time of 40 minutes, at an amplitude of 20%.

### 2.3 Emulsion preparation

Water-in-oil and sugar solution-in-oil emulsions were all prepared at 20% volume fractions. The aqueous phases were added to the oil PMMA dispersion and vortex mixed. For the dodecane system, these were mixed until the oil layer became clear, confirming that essentially all the PMMA had adsorbed onto the water–oil interface. For the tributyrin system, emulsions were instead vortex mixed for 30 s with 30 s rest intervals, for 2 minutes of total mixing time. For these emulsions the oil phase did not become clear; indeed, small particle aggregates could be seen in the confocal microscopy images.

In both systems, after the emulsion fabrication, equal volumes of the sugar solution and pure water emulsions were combined, shaken gently for a few seconds, pipetted into a cavity slide, and immediately observed with a Zeiss LSM 700 confocal microscope. The droplets observed were those that sedimented to the bottom of the coverslip.

### 2.4 Droplet tracking

#### Dodecane system

The dyed components were pure water (sodium fluorescein), water stabilising PMMA (NBD), and sugar solution (rhodamine B). Excitation wavelengths of 488 nm and 555 nm were used to excite the NBD labelled particles and sodium fluorescein, and rhodamine B respectively. This allows clear observation of the water droplets in one channel.

Initially the images were manually thresholded to remove droplet edges allowing closely touching droplets to be separated more easily. The resulting image was thresholded again *via* Otsu's method to separate the droplets from the background. An erosion was then applied, and the resulting image used as seeds for the watershed algorithm for segmenting the droplets. Once segmented, RegionProps^11^ was used to acquire *x*, *y* coordinates of each segment as well as properties such as radius. TrackPy^12^ was then applied to track each droplet using the *x*, *y* coordinates.

Droplets that sediment into the plane of the image were removed as a matter of course as the watershed algorithm only used seeds from the initial frame, thus ignoring large droplets that appear later in the video. Droplets that sedimented during observation, making it appear that they were growing, were also filtered out as this would skew the effects of ripening. To account for this, any droplets that grew more than 10% of their initial size at any point during the video were discarded.

#### Tributyrin system

The dyed components were pure water (pyranine) and tributyrin (Nile Blue) and undyed PMMA was used for stabilising the aqueous droplets to facilitate segmentation of touching droplets. Excitation wavelengths of 405 nm and 555 nm were used for the pyranine and Nile Blue respectively. A similar image processing pipeline was deployed for analysing these droplets with the exception of the initial thresholding, as the utilisation of undyed particles separated droplets already. As water droplets have a tendency to cream, any droplets that appear for a short number of frames compared to the total number of frames were discarded.

## 3 Results and discussion

### 3.1 Stability of emulsions

Comparing the two dispersions of PMMA in dodecane and tributyrin, the PMMA in dodecane is cloudy and sediments within a few days, however, the tributyrin dispersion appears slightly cloudy after months with no sedimentation. This could be indicative of the contrasting particle–particle interactions. The cloudy dodecane dispersion suggests the PMMA may be aggregating slightly, thus scattering light, and the large difference in densities explains the relatively fast sedimentation. Alternatively, in tributyrin there may be reduced PMMA aggregation, resulting in less light scattering. The densities are also more closely matched leading to a reduction in sedimentation.

**Fig. 2:**
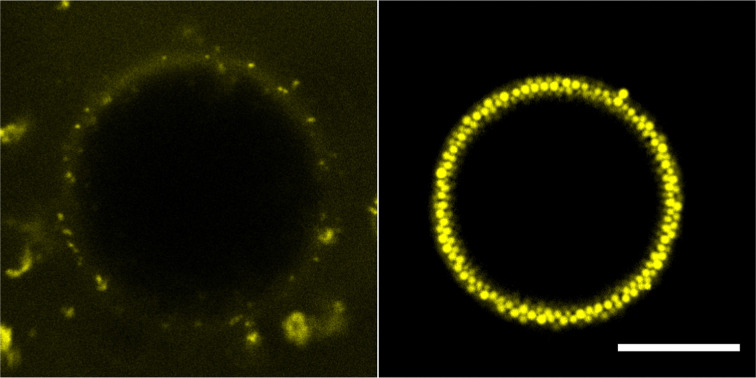
Comparison of a water droplet stabilised by PMMA (*r* ≈ 362 nm) in tributyin (left) and dodecane (right). For the tributyrin system the PMMA colloids sparsely coat the droplet interface and appear to cluster in the continuous phase. In the dodecane system the PMMA forms a dense monolayer on the droplet interface. The scale bar is 10 μm.

Before considering the effects of compositional ripening, the water droplets alone are considered. Fig. 2 shows water droplets of similar size stabilised by PMMA in both dodecane and tributyrin. In dodecane, the water droplet is coated by a dense PMMA monolayer, whereas in tributyrin the droplet is not well coated and the colloids appear to form clusters in the continuous phase. Interestingly for tributyrin, these clusters only form upon the addition of water, suggesting there may be some affinity for water–PMMA interactions. For the dodecane emulsions the droplets clearly sediment within a few minutes and for the tributyrin the droplets cream over the course of an hour. However, both emulsions appear stable after 2 weeks with no visible phase separation; much longer than the timescales considered for the ripening experiments.

For the sugar droplets as the sugar concentration is increased, it appears that qualitatively the droplet sizes become more polydisperse. This is a feature of the initial emulsions which remain stable until they acquire more water during ripening, described below.

### 3.2 Compositional ripening

**Fig. 3:**
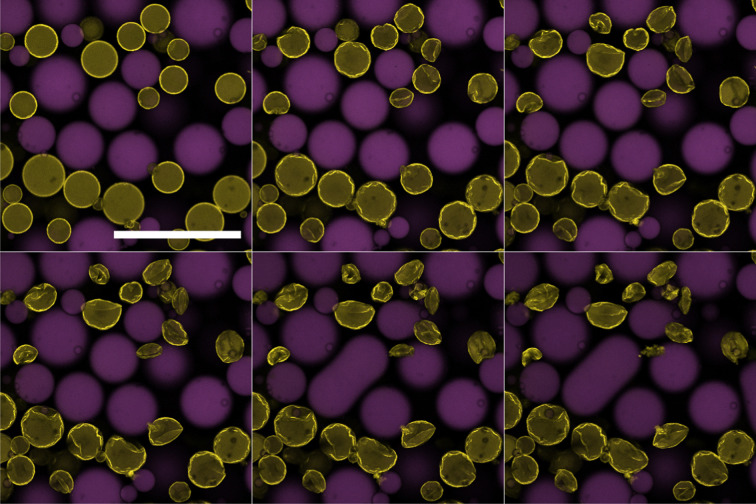
Confocal microscopy time stills of the dodecane system undergoing compositional ripening over 2 hours. Water droplets (yellow) appear to crumple and lose mass while the 64% sugar solution droplets (purple) grow and slowly coalesce. Scale bar of 250 μm.

To be certain of mass transfer in the expected direction the pure water and sugar solution emulsions were combined, and then immediately observed. In this system we consider the pure water droplets as ‘mobile’ and the sugar solution droplets as ‘immobile’, given the relative solubilities of water and sucrose in oil. We begin observations as quickly as possible following mixing of the two populations of droplets. In the dodecane system, two observations confirm the mass transfer out of the water droplets into the sugar droplets (Fig. 3). Firstly, the water droplets crumple. As water diffuses out of the droplets, the interface compresses. Due to the high energy cost of colloid removal, the interface has to buckle. The resulting water droplet morphologies appear similar to those reported previously.^13^ Secondly, the sugar droplets grow and coalesce. As the sugar droplets grow, due to the lack of excess colloids in the continuous phase, areas of the interface will appear that are free of colloids. When such areas of two sugar droplets meet, arrested coalescence^14^ may take place, indicated first by the formation of ‘peanut’ shaped droplets which then become more spherical over time. Prior to coalescence, which complicates tracking, quantitative analysis indicates that the loss of volume of the water droplets is mirrored by the gain of volume of the sugar droplets.

**Fig. 4:**
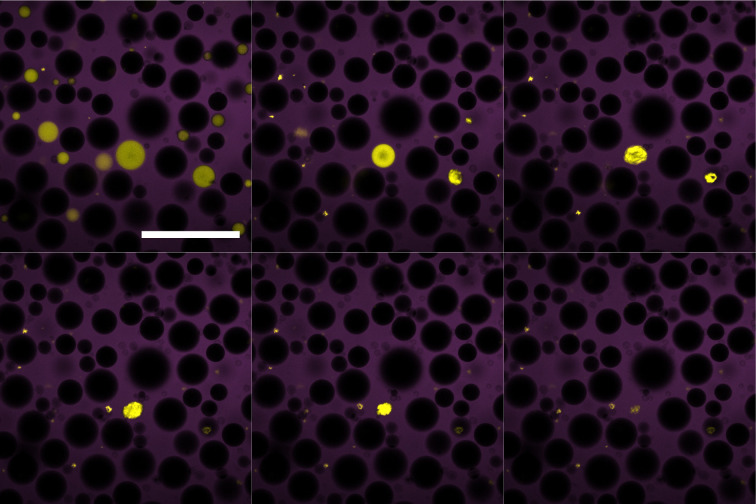
Confocal microscopy time stills of tributyrin system undergoing compositional ripening over 30 minutes with 50% sugar solution droplets. The water droplets (yellow) initially uniformly decrease in size before the interface starts to crumple, reaching stable a size in the form of small clusters. Scale bar of 250 μm.

The tributyrin system evolves in a similar manner to the dodecane system, however at a much faster rate (Fig. 4). This is beneficial as it allows the full ripening process to be observed within a convenient time frame. Given the higher solubility of water in tributyrin, this behaviour is expected. The water droplets appear to undergo an initial uniform decrease in size followed by the crumpling of the interface as shown by the large droplet in Fig. 4. This is in agreement with Fig. 2 in which the droplet is not initially well coated by colloids; only once a sufficient amount of mass transfer takes place, will the interface then crumple. This lack of an initial dense monolayer at the interface may also be an important factor in the faster rate of ripening. The particle clusters observed in the tributyrin system sediment during the ripening process and play no obvious role in water transport. It is important to note that the tributyrin system, although made with the same ratios of water to sugar solution as the dodecane system, has fewer water droplets in the plane of the image. This is due to the creaming of many of these droplets. This may also be a contributing factor to the observed increased rate of ripening.

For both systems coalescence was only observed between the sugar droplets and is further evidenced by two very clear populations with no mixed dye droplets. This is consistent with the particle layer for water droplets becoming increasingly compact, protecting them from coalescence. Sugar droplet coalescence was observed to a lesser extent in the tributyrin system, perhaps due to the smaller number of water droplets in the plane of the image. The driving force for the mass transfer is also assumed to be mainly due to compositional ripening, as the chemical potential arising from concentration differences far outweighs that of the Laplace pressure.

### 3.3 End fate of droplets

**Fig. 5:**
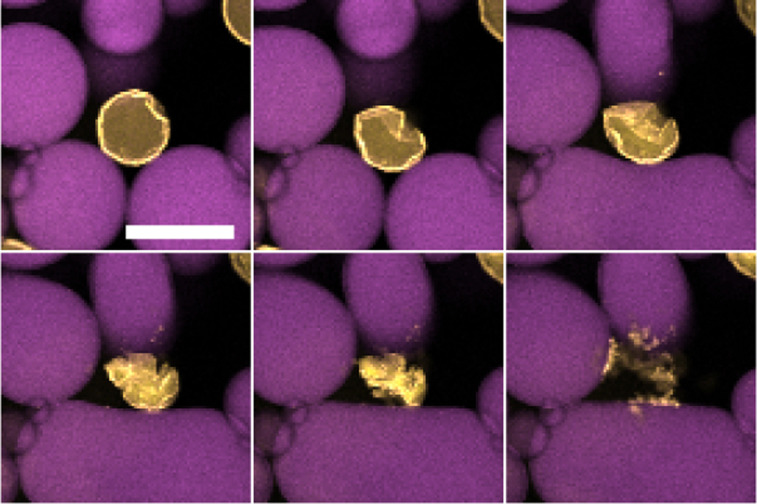
Water droplet (yellow) surrounded by 50% sugar solution droplets (purple) in dodecane undergoing ripening over 2 hours. The droplet initially crumples and after a period of time eventually ‘explodes’ leaving what appears to be clusters of PMMA colloids. Scale bar of 50 μm.

Interestingly the final fate of the droplets between the two systems is different. For the dodecane system, particular droplets that lose sufficient mass appear to eventually ‘explode’. This is shown in Fig. 5 where the droplet crumples into a small structure before the particles appear to be suddenly ejected over a few minutes. This phenomena was found to only occur at high sugar solution concentrations in which the local environment of the droplet was sugar-rich, as well as the droplets being sufficiently small. Larger droplets and those in lower sugar solution environments were simply not observed for a long enough duration to determine whether their fate was the same. Indicative calculations confirm that there remains a substantial chemical potential gradient at the time when droplets are typically exploding.

There are some possible explanations for this ‘exploding’ behaviour. The colloids are sterically stabilised in dodecane so when their separation is reduced on the interface they remain repulsive. The wetting angle of PMMA at the water–dodecane interface is quite extreme so delamination is likely to be easier. The fact that these ‘explosions’ are not a gradual process and occur on the timescales of a few minutes, may suggest this is not the ejection of individual particles but rather larger structural components. As a result, these ‘explosions’ may be related to the ejection of folds^15^ or delamination.^16^ This phenomena may also be related to the various morphological fates of bubbles undergoing dissolution for various particle-to-bubble ratios.^17^

**Fig. 6:**
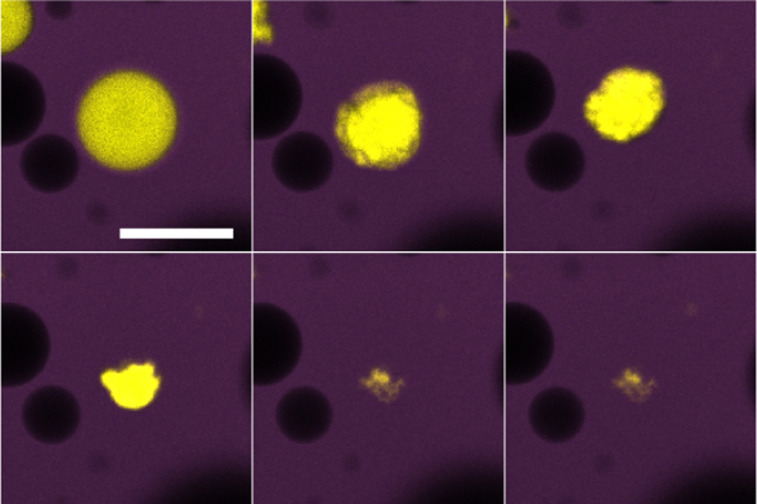
Water droplet (yellow) in tributyrin (purple) surrounded by 64% sugar solution droplets (black) undergoing ripening over 10 minutes. The droplet rapidly loses water and buckles until it reaches a stable shape. Scale bar of 50 μm.

**Fig. 7:**
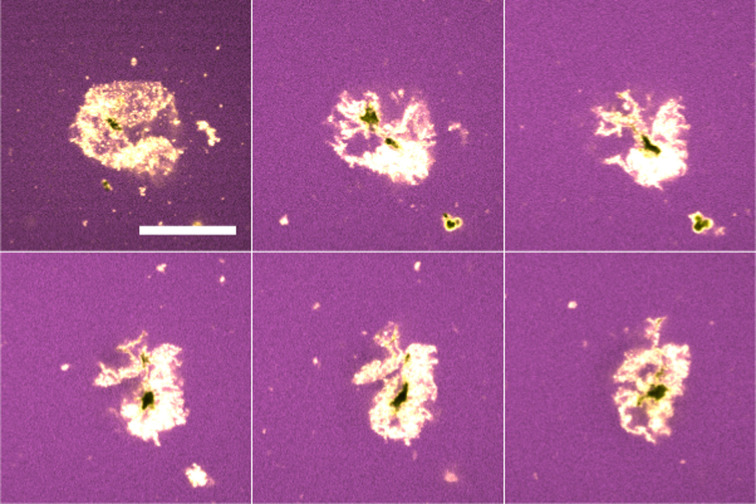
Stills through the *z*-direction of a crumpled water droplet in tributyrin (blue) stabilised with PMMA (yellow). In this case, the water was left undyed and thus appears black. These stills were taken 90 minutes after initialising the ripening – long after the droplet size plateaus. The black regions suggest some water still remains. Scale bar of 15 μm.

In contrast to the dodecane system, water droplets in the tributyrin system appear to crumple as they decrease in size and then attain a stationary jammed shape (Fig. 6), similar to structures in Erni *et al.*^18^ where they are referred to as ‘ghosts’. Z-stack micrographs of crumpled droplets were performed after their size plateau, and indicate that there may be some water retained in the centres of these crumpled states (Fig. 7). The observation of PMMA clusters in tributyrin following the addition of water suggests that the particle–particle attractions become significant in the emulsion environment. The final crumpled states in tributyrin may reflect this key difference between the tributyrin system and the observations we have described in dodecane.

### 3.4 Quantitative results

The concentration of sugar solutions in the ‘immobile’ droplets was varied to control the driving force for ripening. From eqn (2), one would expect that increasing the sugar concentration, increases the difference in chemical potential between both droplet species, thus increasing the rate of ripening.

**Fig. 8:**
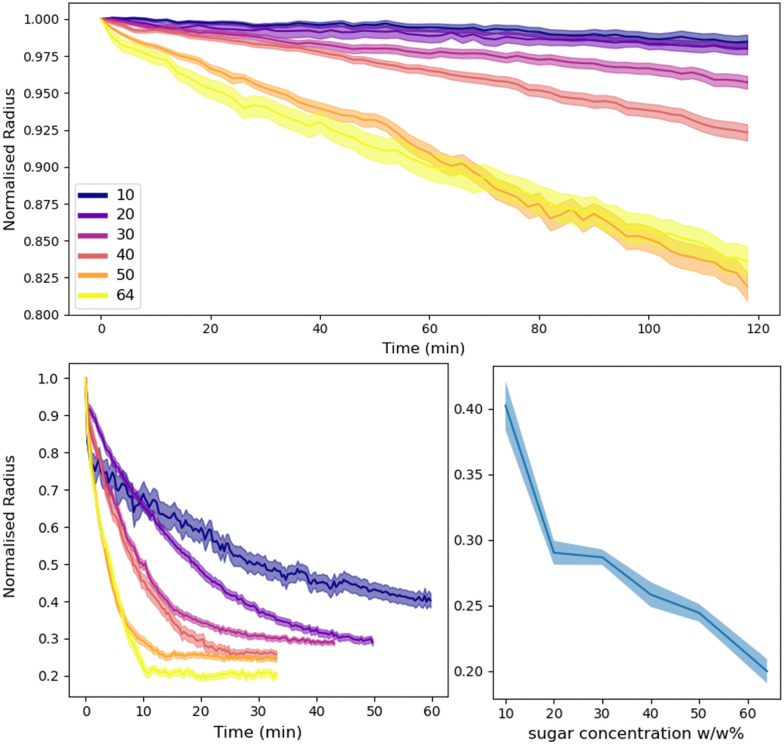
Average normalised water droplet radius with time for various sugar concentrations in the dodecane system (top) and tributyrin system (bottom left). Bottom right shows the final normalised droplet size as a function of sugar concentration for tributyrin.

Sugar concentrations from 10–64% by weight were prepared for the dodecane and tributyrin emulsions. These were then combined with the respective pure water emulsions and observed with confocal microscopy and analysed with the image processing pipeline outlined in Section 2.4. Fig. 8 shows the water droplet evolution for both systems. On the graphs are the normalised radius *versus* time, where the normalised radius is the droplet radius divided by its initial size. An exploration of the role of the local droplet environment is presented in the ESI.† We find that, while the total sugar concentration and the initial water droplet size dominate the ripening rate, the local sugar environment close to a particular water droplet is also influential.

In the dodecane system, the ripening takes many hours to complete and thus was difficult to track to completion as drift during recording would cause droplets to fall in and out of focus, skewing the measurements. Nonetheless, we can clearly see that increasing the sugar concentration increases the rate of ripening. At 64% and 50% sugar fractions there appears to be some crossover. This is believed to be due to a combination of a small sampling size and a large variation of the environment of the droplets *i.e.* the local size and area fraction of droplets. Additionally, due to smaller droplets ‘exploding’ at these concentrations the average radius can also become skewed. At lower concentrations of 10% there appears to be little to no change over these timescales, however over the course of a day or more, there is sufficient ripening and the particle coverage fails to prevent the mass transfer from occurring.

The tributyrin system behaves in a similar manner to that seen with dodecane however on a much faster timescale, thus allowing us to observe the ripening process to completion. In addition, the crumpling droplets eventually remain at a stationary size, resulting in the plateauing of the droplet radius as seen in Fig. 8. The increase in ripening reflects the different solubilities of water in the two oils and the lack of monolayer at the interface. Interestingly, as shown in Fig. 8 the final size of the crumpled states appear to have some dependency on the concentration of sugar solutions, with lower sugar concentrations leaving larger structures. Indicative calculations confirm that there remains a substantial chemical potential gradient at the time when droplets are trapped in their final crumpled state. Assuming water droplets come from a similar population distribution, one might expect that after all the water is driven out of the droplets, the size of the crumpled states would be the same for all sugar concentrations. This, with the results from Section 3.3, may indicate that perhaps once the PMMA coating is sufficiently jammed, the rheological properties are able to overcome the water migration. Alternatively, simple variations in the initial droplet sizes could be behind this observation and should be investigated further. This is an important result as it potentially shows that by simply manipulating Pickering emulsions, compositional ripening can be suppressed without the need of many components, or a continuous shell.^7^ This potentially opens up routes, using bio-compatible Pickering emulsions, to suppress the effects of ripening in edible emulsions.

## 4 Conclusion

Here we studied particle-stabilised water-in-oil Pickering emulsions undergoing compositional ripening, with the driving force being the addition of sugar solution droplets. Dodecane was used as the oil phase in a model system leading to the formation of a dense PMMA monolayer around the water droplets. In our second system, dodecane was replaced with tributyrin. This oil has a higher water solubility and modifies the behaviour of the colloids on the liquid–liquid interface. As expected, using tributyrin resulted in a much faster rate of ripening.

The concentration of sugar in the droplets was varied in order to increase the compositional ripening effect. For both the water-in-dodecane and water-in-tributyrin emulsions the rate of ripening increased with increasing sugar concentration, suggesting that the interfacial coating of particles was not playing a dominant role compared to the osmotic pressure.

Confocal microscopy has revealed contrasting end fates for the two systems. The droplets in dodecane ‘explode’ whereas in tributyrin they crumple into stationary shapes. Unexpectedly, the crumpled structures left from the tributyrin system appear to have some size dependency on the sugar solution concentrations. Upon further investigation, it appears these structures may retain water after the majority of the mass has been lost. The exact mechanisms behind this are the subject of future exploration.

In this work we have shown that the simple act of modifying the continuous phase from dodecane to tributyrin leads to an assortment of emulsion changes. Together they lead to contrasting evolutions of the emulsions, and provide us with possible solutions to overcome compositional ripening in simple Pickering systems. When considering water-in-oil emulsions in the context of low calorie foods, complex interfaces will inevitably play a role due to the complex landscape of food and should be investigated.

## Author contributions

RT carried out the investigation and performed the formal analysis. Supervision was by PSC, BG and TC. The project conceptualization was by BG, TC and PSC. Writing – original draft was by RT. Writing – reviewing and editing were carried out by all authors.

## Conflicts of interest

There are no conflicts to declare.

## Supplementary Material

SM-019-D3SM00856H-s001
