# Supplementary material for: Long term water trapping in Pickering emulsions undergoing compositional ripening
Source: Soft Matter. 2023 Nov 9;19(48):9428–34. doi: 10.1039/d3sm00856h (PMC10718171; doi:10.1039/d3sm00856h)
Supplement: SM-019-D3SM00856H-s001 [file SM-019-D3SM00856H-s001.pdf]

Electronic Supplementary Material (ESI) for Soft Matter.
This journal is © The Royal Society of Chemistry 2023

Electronic Supplementary Information: Long term water trapping in Pickering emulsions undergoing compositional ripening

Raj Tadi*, Beth Green‡, Thomas Curwen‡, and Paul S. Clegg*

October 31, 2023

1 Quantitative influence of droplet environment

The concentration of sugar droplets clearly dictates the rate of ripening of water droplets, as expected from theory. When observing the videos more closely it appears that certain droplets may not strictly obey this trend. One would expect that larger water droplets ripen slower, however the local environment appears to also play a role. As illustrated in Fig. 1, the water droplets in the 50% sugar concentration sample ripen marginally faster than those in the 64% sample, even though the sizes are similar. These two environments are quite different, with the droplet surrounded by more sugar droplets ripening faster.

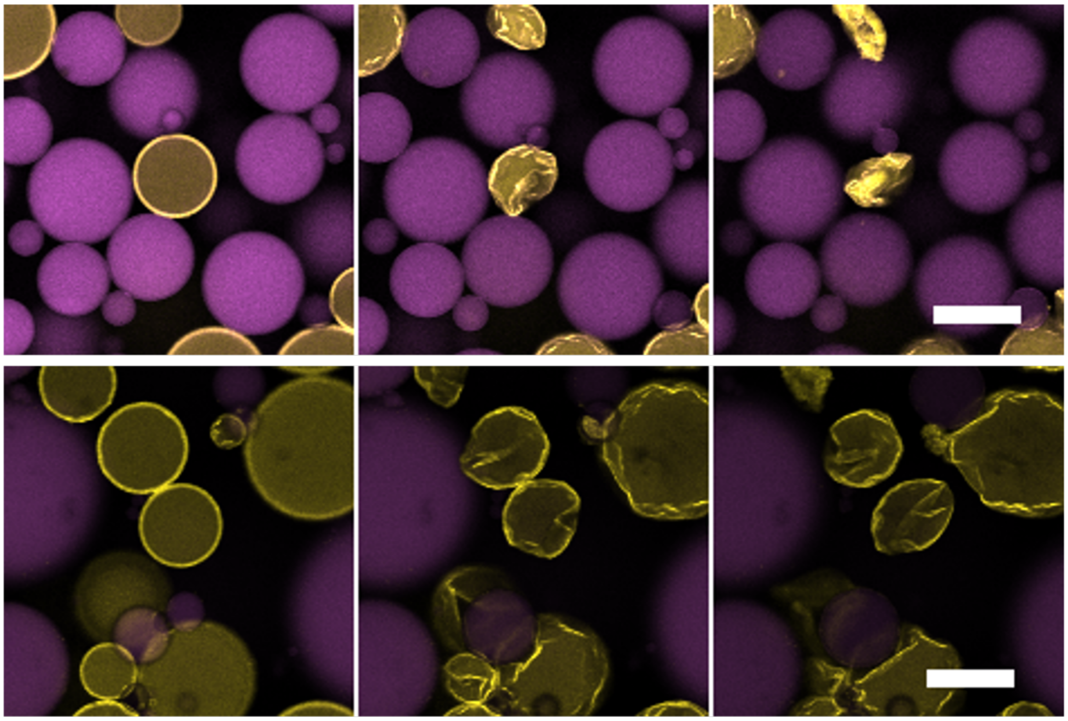

Figure 1: Example of two water droplets of similar size ripening in dodecane at 50% (top) and 64% (bottom) sugar concentrations over 2 hours. In this example the lower concentration droplet ripens faster, illustrating that other factors can play a role, such as the local environment . Scale bar 50 μm.

[0]* School of Physics and Astronomy, University of Edinburgh, Peter Guthrie Tait Road, Edinburgh, EH9 3FD, UK
[0]‡ Mondelēz International, Reading Science Centre, Whiteknights Campus, Pepper Lane, Reading, RG6 6LA, U.K

1.1 Area fraction

One method to quantify the environment of the water droplets is to look at the area fraction surrounding the droplets that is occupied by water and sugar. For each water droplet a circular region is drawn from the edge of the droplet with a given radius. The area occupied by sugar and other water droplets inside this region can then be calculated.

This is visualised in Fig. 2, where the local area fraction for water (left) and sugar (right) is analysed for each water droplet. It is seen that water rich regions in (yellow) roughly correspond to low sugar regions (purple) and vice versa.

Additionally varying the size of the local region and observing changes in the area fractions can be explored (Fig. 2). As expected, the area fractions approach their global values as the radius is increased in addition to the standard error decreasing.

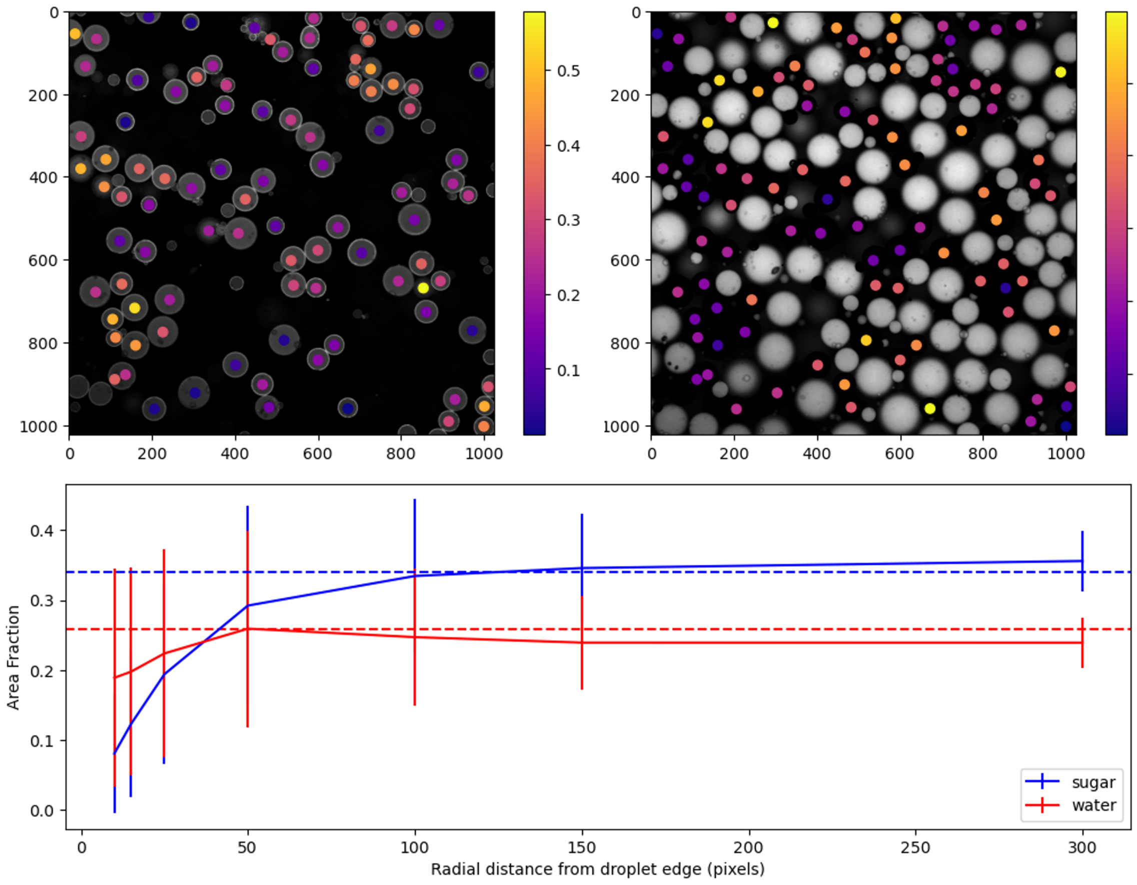

Figure 2: Top: Area fraction of water (left) and sugar (right) surrounding water droplets within a radius of 50 pixels, with the colour bar giving the corresponding value. Bottom: The average area fraction of the water and sugar as the radial distance of the region is increased. As the radius is increased the average approaches the area fractions of the entire images.

1.2 Machine learning

A combination of local descriptors and whole sample descriptors can be used to train a machine learning model in order to predict the final droplet size at a later time. The quantitative parameters are the initial droplet size, the total sugar concentration and the area fraction of water and sugar within different distances of the droplet. The importance of any of these features to the model can also be compared to qualitative observations.

Here, the machine learning model of choice is the Random Forest. This class of model is made by pooling results from very many separate Decision Trees each of which only see a subset of the

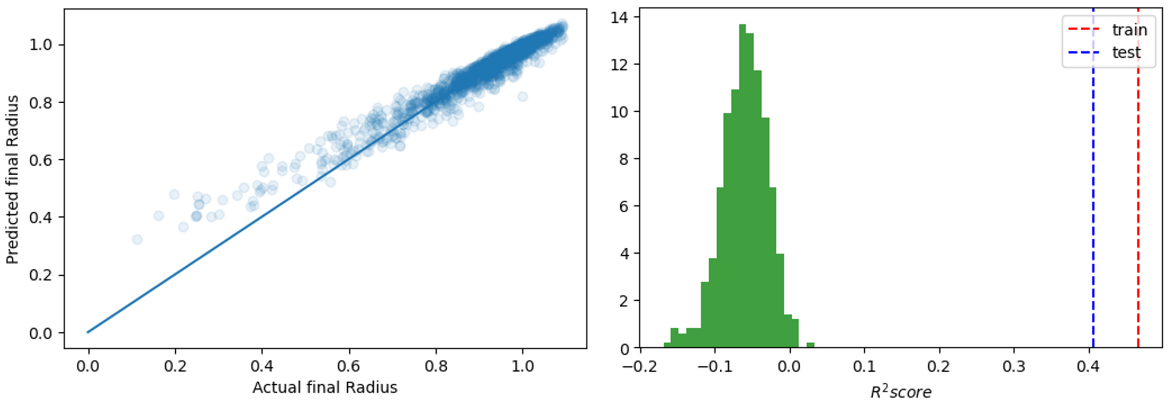

Figure 3: Left: Plots of the actual final normalised radii against the predicted values from the best performing Random Forest regressor. Perfect predictions would lie on the diagonal line, however the regressor makes predictions that are systematically too high. Right: Histogram of the R^2 score from 1000 fits of the best Random Forest regressor on shuffled data.

descriptors. Random Forests come in-built with a relative feature importance capability. This can provide an idea of which of the descriptors play a large role in controlling ripening. Our dataset is split into test and training sets of ratio 1:4. Using GridSearchCV the model's hyperparameters were tuned to determine the best performing model with respect to an R^2 score. Using the optimal hyperparameters the model is fitted to the testing dataset. The best model provided a training score of 0.47 and a test score of 0.41. This relatively low score may be indicative of much more information being required for a successful prediction of the emulsion evolution i.e droplets outside and above the field of view. To establish whether this score is significant, the dataset is randomised such that the final radii should have little correlation with the descriptors. The shuffled dataset is then used for training on the best Random Forest model and test scores determined. This process is repeated 1000 times and the results shown in Fig. 3. Here it can be seen that the random forest performance on the properly labelled data is far greater than the shuffled, confirming that the model is not just fitting to noise.

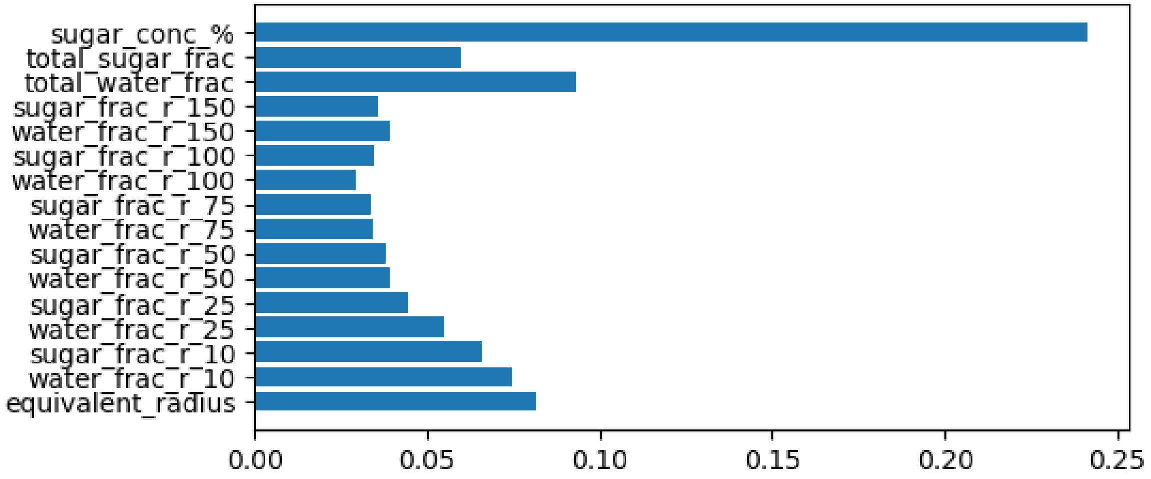

Figure 4: Feature importances from Random Forest regressor trained for predicting the final normalised radius. The values of r correspond to pixel radii from the droplet surface.

To further visualise how well the model is performing, a predicted against actual value plots can be made (Fig. 3). From this it can be seen that many points do lie close to the true value, but as the final actual normalised radius is decreased the predicted value is often higher. This could be due to the fact

that droplets eventually ‘explode’ which is not taken into consideration in this approach. Additionally there may simply not be enough data points at the lower final raiii for the machine learning model to be working effectively.

Using the best performing model, the feature relative importances can be determined, illustrated in Fig. 4. As expected the concentration of the sugar solution used and the initial size of the water droplets play a large role. The total area fractions play important roles too and this is attributed to the fact that the mixing of the emulsions is not perfect and they do not sediment evenly, as a result the final emulsion composition when being observed may not perfectly reflect the prepared composition. Those samples in which there are more sugar droplets are therefore expected to ripen faster.

Looking at the importance of the local area fraction parameters there is an informative trend. As the size of the region around the droplet is increased, the importance of the parameter decreases. This may be indicative of a proximity effect, whereby the local environment has a larger role to play.
